# The overlooked amoebae of an agroecosystem of black soil land in China: five new species of dictyostelids

**DOI:** 10.1128/aem.00025-25

**Published:** 2025-06-10

**Authors:** Zhaojuan Zhang, Jialing Jin, Yuqing Sun, Yingbai Wang, Jing Zhao, Songning Guo, Yue Zou, Wenhan Chen, Steven L. Stephenson, Yu Li, Junzhi Qiu, Pu Liu

**Affiliations:** 1 Engineering Research Center of Edible and Medicinal Fungi, Ministry of Education, Jilin Agricultural University 85112, Changchun, China; 2 Department of Biological Sciences, University of Arkansas 3341 , Fayetteville, Arkansas, USA; 3 State Key Laboratory of Ecological Pest Control for Fujian and Taiwan Crops, College of Life Sciences, Fujian Agriculture and Forestry University 528652, Fuzhou, China; Colorado School of Mines, Golden, Colorado, USA

**Keywords:** cellular slime molds, Amoebozoa, plant-associated protists, farmland, black soil region, five new taxa

## Abstract

China’s northeastern black soil region, a global black soil hotspot, is vital for supporting national food security with its fertile and agricultural-friendly soil. The dictyostelids are well-known eukaryotic microorganisms with an unusual multicellular stage in their life cycle, and they inhabit surface soils worldwide. Although numerous species have been isolated from forest ecosystems and their remarkable biological activities have been reported, species of plant-associated dictyostelids of agroecosystems remain surprisingly understudied, especially in the agroecosystems of the black soil region of Jilin Province. In this study, the diversity of this group was evaluated based on corn, cabbage, and soybean soil samples from black soil land, including mollisol and chernozem soil types in Jilin Province of China. Morphological characterization and molecular analyses of the nuclear small subunit (18S) were used for species delineation. A total of 20 strains of dictyostelids were isolated from four localities, and on the basis of morphological examination and molecular phylogenetic analyses, five species of dictyostelids are described as new to science. These are *Coremiostelium viridiflava*, *Raperostelium macrosorus*, *Dictyostelium torta*, *Cavenderia densissima*, and *Polysphondylium sparsiramus*. These new species are described in detail, with morphological and molecular distinctions from their closest relatives explicitly discussed. This study not only revealed the diversity of dictyostelids in agroecosystems but also provided more research data for further studies in black soil land microbe protection in China.

**IMPORTANCE:**

Despite the recognition of the presence of dictyostelids in various ecosystems, only limited information is available on their diversity and characteristics, particularly in the agroecosystems of China’s northeastern black soil region. These eukaryotic microorganisms are known for their multicellular life cycle stages and widespread occurrence in surface soils. However, the species of dictyostelid in the black soil region of Jilin Province remain largely unexplored. In this study, a total of 20 dictyostelid strains, including five newly described species, were isolated and characterized from soil samples. Morphological examination and molecular phylogenetic analysis were conducted to document these new species. This research not only revealed the diversity of dictyostelid species in this important agroecosystem but also provided valuable data for future studies on microbe protection in China’s black soil land. The discovery and detailed analysis of these new species contribute to our understanding of dictyostelid ecology, biology, and potential roles in agroecosystems.

## INTRODUCTION

Soil microorganisms constitute a pivotal component of soil ecosystems, with their diversity and community composition forming the cornerstone upon which agricultural productivity relies (1–3). Black soil land, characterized by its excellent properties and high fertility (4), serves as a crucial farmland resource, especially in Jilin Province, Northeast China (including Inner Mongolia), and also nationwide. The ecological significance of black soils lies not only in its physicochemical properties (e.g., high organic carbon content and neutral pH) but also in its capacity to support diverse microbial communities that drive nutrient cycling (5, 6). Black soil encompasses six distinct categories: mollisol, chernozem, meadow soil, albic soil, dark brown soil, and brown soil (7, 8). These soil subtypes create heterogeneous microhabitats through variations in moisture retention, organic carbon availability, and microbial activity—factors that may critically influence protist community assemblages.

The fungal and bacterial community diversity and composition in black soils of Northeast China are significantly influenced by contemporary soil characteristics such as carbon content and pH, with long-term chemical fertilization further reducing bacterial biodiversity and abundance, particularly at higher fertilizer concentrations (9, 10). This decline in bacterial diversity may cascade to higher trophic levels, potentially diminishing the populations of bacterivorous protists such as dictyostelids that regulate microbial loop dynamics.

However, surprisingly little research has been conducted on the plant-associated protist assemblages within black soil ecosystems. Notably, the interactions between soil physicochemical heterogeneity (e.g., among black soil subtypes) and protist functional diversity remain unexplored despite their implications for agroecosystem resilience. It is well-recognized that the plant-associated microbiome constitutes a crucial component for sustaining biodiversity and ecosystem productivity while also playing a vital role in crop growth, nutrient acquisition, resistance to pathogens, and tolerance to abiotic stresses in the context of global change (11, 12). Soil protists, as significant predators of bacteria and fungi and ubiquitous contributors to nutrient cycling and energy transfer, largely remain an unexplored “black box” (13–16). In black soils, where agricultural intensification alters microbial communities, understanding protist diversity becomes essential for predicting ecosystem responses to land management.

The dictyostelids, a group of terrestrial protists that emerged within the Amoebozoa lineage several hundred million years ago, evolved multicellularity to survive starvation by dispersing as aerially borne spores supported by dead stalk cells (17–19). As bacterial predators, they are crucial for soil nutrient recycling and nutrient-mineral turnover (20–22). Their trophic role may be particularly pronounced in fertile black soils, where high bacterial biomass could sustain diverse dictyostelid communities. However, the relationship between black soil subtypes (e.g., mollisol vs albic soil) and dictyostelid niche partitioning remains unknown. To date, the group is comprised of approximately 176 species worldwide (Index Fungorum: http://www. indexfungorum.org).

Dictyostelids have traditionally been classified by morphological characteristics into three genera: *Dictyostelium* (unbranched/irregular sporocarps), *Polysphondylium* (wheel-like branched sporocarps), and *Acytostelium* (non-cellular sporocarp stalks) (23, 24). Among these characteristics (Table 1), the growth habit and branching pattern of the sorocarp appears to be diagnostic for distinguishing species (25). However, morphological variation is often observed as depending upon the growth substrate and incubation duration (23, 26), resulting in an expanded phylogeny that underscores an enhanced morphological plasticity at deeper taxonomic hierarchies (27). As such, species identification based on morphological characteristics is problematic and impractical. To address this problem in the present study, DNA sequences of the small subunit ribosomal RNA (18S rRNA) were employed to delimit and recognize dictyostelid species and infer their phylogenetic relationships (28).

**TABLE 1:** Key morphological terms in dictyostelid biology

Term	Definition
Sporocarp	Closed, sac-like fruiting body where spores are enclosed within a protective covering (peridium)
Sorocarp	Open fruiting bodies consisting of a stalk (sorophore) supporting one or more spore masses (sori)
Sorophore	Vertical stalk-like structure composed of vacuolated cells
Sori	Terminal spherical clusters of spores at the apex of sorocarps or branches of sorocarps
Sorogen	The developing sorocarp structure during morphogenesis
Spore	Reproductive units with resistant coats, enabling survival in harsh environments
Aggregation	Multicellular clusters formed by chemotactic aggregation of starving amoebae
Pseudoplasmodium	Migratory slug-shaped multicellular structure (also called “slug”)

Dictyostelids have been reported as nature’s inventors of multicellularity (29, 30). They are commonly isolated from various terrestrial microhabitats such as animal dung (31) and the mantle of dead organic matter “canopy soil” (32) associated with epiphytes. Dictyostelids are especially common in forests (33–35). Furthermore, they have been isolated from various agroecosystems, including agricultural soils, farmland, and experimental plots (36–41) (Table S1). However, only four species of dictyostelids have previously been reported from agroecosystems in China. These are *D. giganteum*, *D. sphaerocephalum*, *D. mucoroides*, and *Cavenderia aureostipes* (40, 41). Many agroecosystem species remain unidentified owing to the lack of resolution in phylogenies and the paucity of morphological characteristics. The purpose of this study was to investigate plant-associated dictyostelids associated with black soil in Jilin Province in China and to identify them using morphological characteristics and phylogenies (18S).

## MATERIALS AND METHODS

### Collection sites and field sampling

The new species were isolated from farmland habitats across four localities and three cities of Jilin Province. These were Changchun City (Dongwa community and Huanzidong Village from Gongzhuling City), Liaoyuan City (Dongliao County), and Songyuan City (Xincheng Town from Ningjiang District). Detailed information about the soil samples, including soil nos., crops, associated soil characteristics (soil types and sampling depth), geographical location, and weather conditions is provided in Table S2.

**Fig 1:**
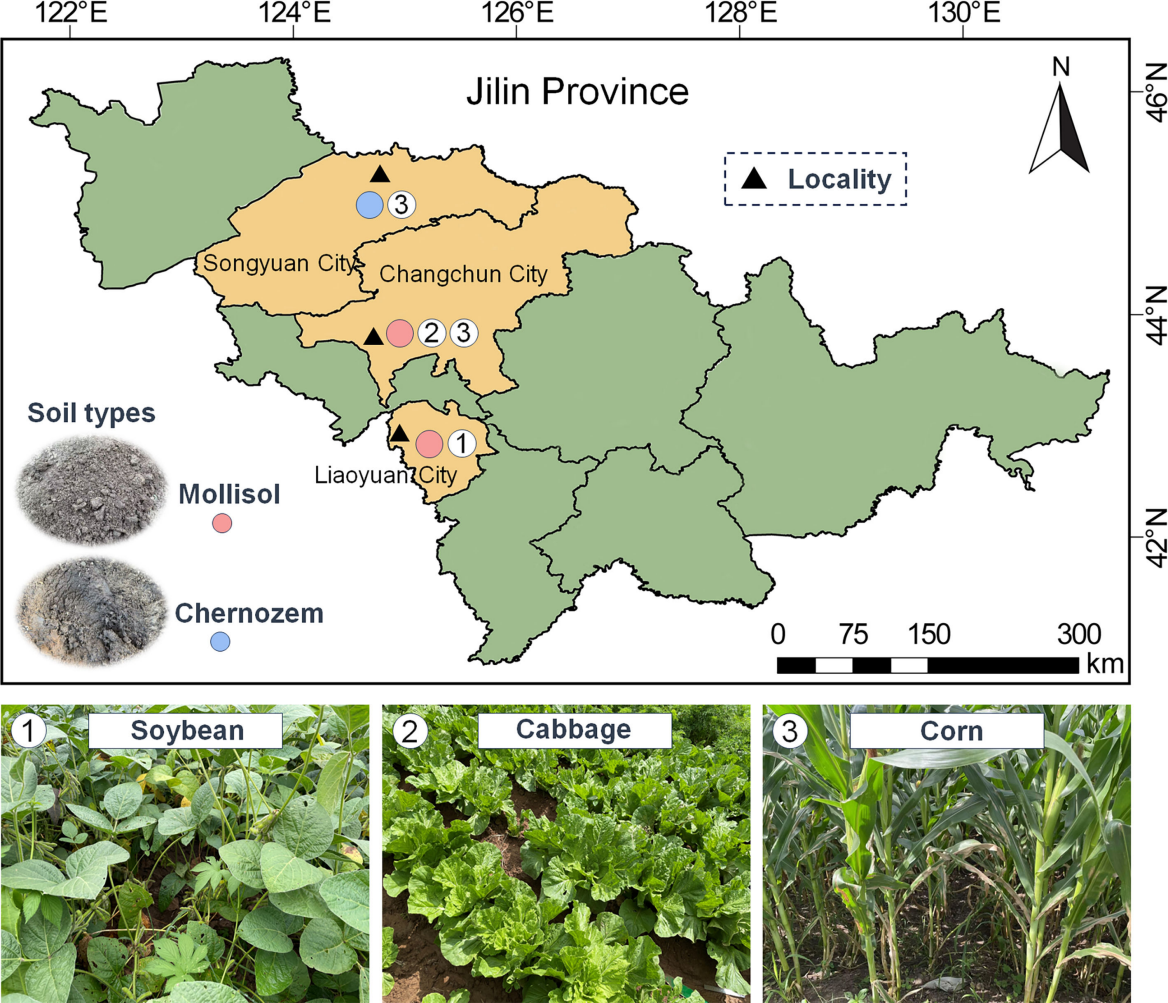
Study area and the sampling sites in the agroecosystem of black soil lands in Jilin Province, China. The map was drawn using ArcGIS (42) software. The details of the samples (e.g., soil no., geographical location, crops, associated soil characteristics) are shown in Table S2.

Eight soil samples were collected from four localities (L1–L4) in Jilin Province, encompassing three crop types (corn, cabbage, and soybean) and two soil types (mollisol and chernozem) in August 2022 and 2023 (Fig. 1; Table S2). L1 includes corn and black soil; L2 includes cabbage and black soil; L3 includes soybean and black soil; and L4 includes corn and chernozem. Each sample consisted of 100 g soil from three distinct depths (0–5 cm, 5–10 cm, and 10–15 cm) based on nine-point sampling and was placed in a sterile Whirl-Pak plastic bag. Within a few hours after collection, all samples were transported to the laboratory. The samples were numbered and recorded in the laboratory soil sample database and then stored at 4℃.

### Isolation and cultivation

The isolation techniques utilized in this study followed those described by Cavender and Raper (43). Each sample was meticulously weighed, and an appropriate amount of double-distilled water (ddH_2_O) was introduced to achieve an initial dilution ratio of 1:10. This diluted mixture was vigorously agitated to disaggregate soil particles and suspend amoebae, microcysts, and spores of dictyostelids. Subsequently, a 0.5 mL aliquot of this diluted suspension, accompanied by 0.4 mL of *Escherichia coli* (acting as a nutrient source), was dispensed into six replicate culture plates prepared with hay infusion agar. These plates were then incubated at 23°C under a controlled photoperiod of 12 h of light followed by 12 h of darkness.

For a duration of 2 weeks after the initial appearance of aggregations, each plate was meticulously examined at least once daily. Each isolate retrieved from a plate underwent purification and was cultivated for the purposes of taxonomic analysis and preservation on non-nutrient water agar plates that had been pre-inoculated with *E. coli* and grown for a period of 12–24 h. The spores harvested from these plates were cryopreserved in HL5 medium (44) and stored at −80°C within the herbarium of the Mycological Institute of Jilin Agricultural University (HMJAU), Changchun, China.

### Morphological analyses

Morphological features throughout the life cycle were described as proposed by Hagiwara (25) and Raper (23). Under a stereomicroscope, detailed observations were made on the isolates, and their morphological features, including growth patterns, branching architecture, coloration, sorocarp properties, and dimensions/configurations of aggregates and pseudoplasmodia, were documented. These observations were facilitated by a fluorescent stereomicroscope (Leica M165FC, Germany).

Subsequently, under a dissecting microscope, a vigorous dictyostelid isolate was chosen, mounted on a slide with sterile water, covered, and examined under an optical microscope. The microscopic attributes noted were spore dimensions and shape, the presence of polar granules, sorophores, cell arrangement, and the apical/basal regions of sorophores. These attributes were visualized using a Zeiss light microscope (Axio Imager A2, Germany) and equipped with an ocular magnification of 10× and objective lenses of 10×, 40×, and 100× (oil immersion). Photographic documentation was achieved using a Zeiss AxioCam 506 color microscope camera.

To accurately identify the species isolated in this study, we conducted a comparative analysis of the recorded data and photographic images of the dictyostelids against the morphological characteristics archived in the original literature, which was referenced in Index Fungorum (45) (accessible at http://www.indexfungorum.org/names/Names.asp).

### DNA isolation, PCR amplification, sequencing, and phylogenetic analysis

The molecular protocols followed were those described by Liu et al. (46). Spores from individual isolates were harvested using sterile tips and subsequently integrated with lysis buffer supplied by the MiniBEST universal genomic DNA extraction kit version 5.0 (TaKaRa Bio Inc., Kusatsu, Japan), following the manufacturer’s guidelines. The resultant genomic DNA solution was directly utilized for PCR amplification with primers 18S-FA (5′-AACCTGGTTGATCCTGCCAG-3′) and 18S-RB (5′-TGATCCTTCTGCAGGTTCAC-3′) (47). The PCR mixture, totaling 25 µL, comprised 12.5 µL of Premix Taq (Ex Taq Version 2.0), 1 µL each of forward and reverse primers, 2 µL of template genomic DNA (10 ng/µL), and 8.5 µL of ddH_2_O. Visualization and comparison of PCR products were conducted on agarose electrophoresis gels. The PCR products were sequenced via Sanger sequencing (Applied Biosystems 3730XL) by Sangon Bio-tech Co., Ltd. (Shanghai, PR China). Resultant chromatograms were manually inspected using BioEdit v7.0.9.0 to assess read quality, particularly at sequence termini. Low-quality regions (Phred score <20) were trimmed, and ambiguous bases (e.g., overlapping peaks) were resolved by re-amplification and re-sequencing. Consensus sequences were generated from forward and reverse reads to ensure accuracy. Following verification, the resultant sequences were submitted to the GenBank database. To elucidate their phylogenetic affiliations within the group, all sequences pertaining to related species were retrieved from GenBank for comprehensive phylogenetic analysis (Table S3).

All small subunit ribosomal RNA (SSU) sequences were aligned and individually compared using the ClustalW Multiple alignment algorithm (48), followed by manual adjustments in BioEdit software version 7.0.9.0 (49). Maximum likelihood (ML) analyses were conducted using IQTREE version 1.6.12 (50), employing 1,000 ultrafast bootstrap replicates to ascertain node support values with the “-bb 1,000” parameter, and further refined through hill-climbing nearest-neighbor interchange (NNI) with the “-bnni” option (51). In addition, the SH-aLRT test was applied to ascertain the topological confidence limits with the “-alrt 1000” parameter (52). The “-nt AUTO” parameter was utilized to automatically ascertain the optimal number of cores based on the given data set. ModelFinder, integrated within IQTREE, was used to ascertain the optimal substitution model according to Bayesian information criteria (BIC) (53). For the comprehensive phylogenetic tree, *Physarum polycephalum* (accession no. X13160.1/NC_002508.1), a myxomycete sequence, was designated as the outgroup using the “-o X13160.1” or “-o NC_002508.1” options. In subgroup analyses targeting specific taxonomic divisions, phylogenetically proximal species were selected as outgroups to enhance topological resolution within respective clades. The final trees were visually enhanced using iTOL (54) for display, annotation, and manipulation.

## RESULTS

A total of 20 strains of dictyostelids were identified, and these represented five new species. Of these strains, four were isolated from mollisol (corn, cabbage, and soybean) and 16 from chernozem (corn). The five new species were *Dictyostelium torta* (1 strain), *Polysphondylium sparsiramus* (11 strains), *Raperostelium macrosorus* (1 strain), *Coremiostelium viridiflava* (1 strain), and *Cavenderia densissima* (6 strains) (Table S2).

### Phylogeny

**Fig 2:**
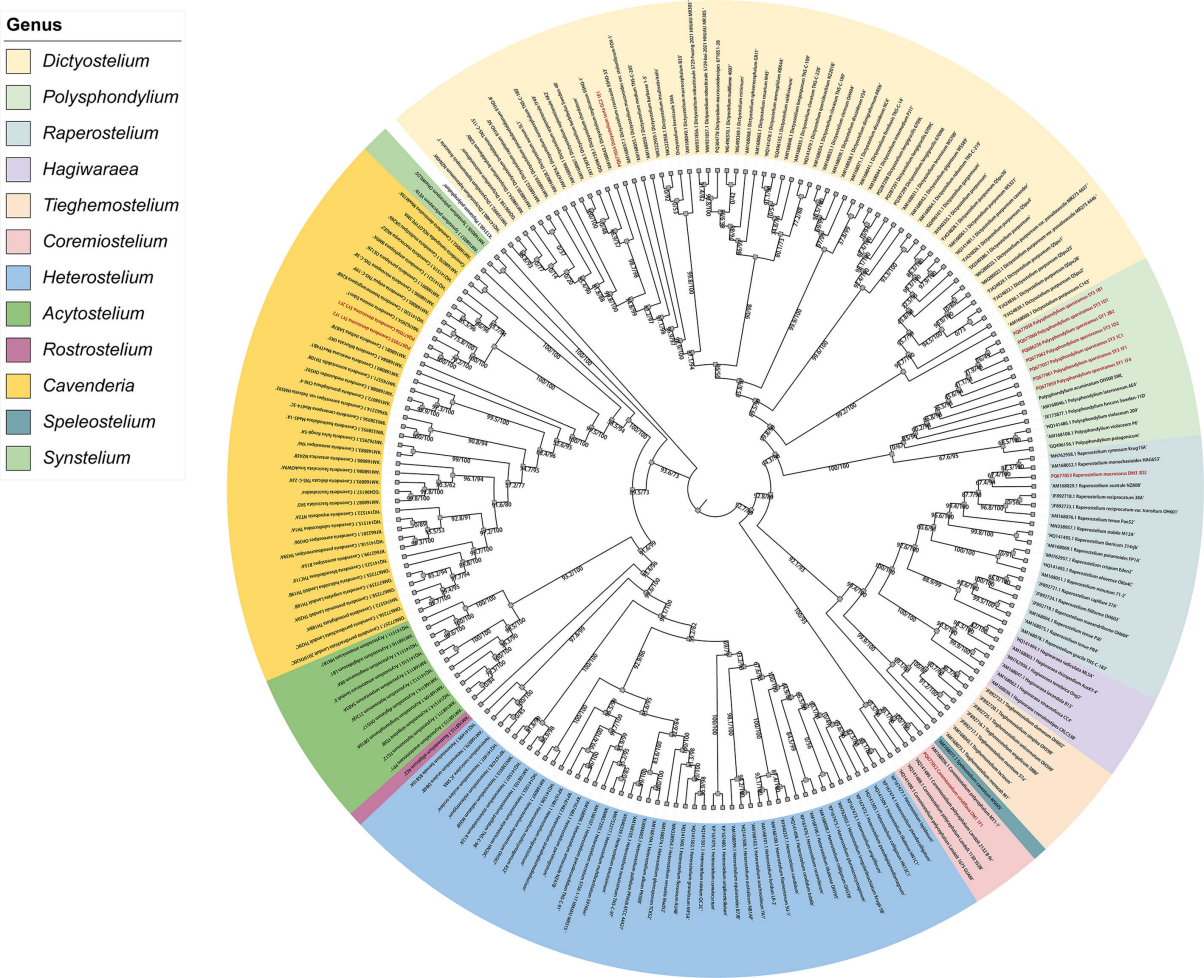
Phylogeny of all known species of dictyostelids based on SSU rDNA sequence data. Newly generated sequences are indicated in red.

There were 212 strains from 168 dictyostelid species were included in the phylogenetic analyses with the outgroup species *Physarum polycephalum*. Twelve SSU sequences were newly generated in this study, which were from 11 strains from Jilin Province, China. The phylogenetic tree of Maximum Likelihood (ML) was constructed based on SSU data, which comprised a total of 1,600–1,800 base pairs (bp). The ML tree within 12 genera is presented in Fig. 2 with SH-aLRT and ultrafast bootstrap support (%) indicated on the branches.

**Fig 3:**
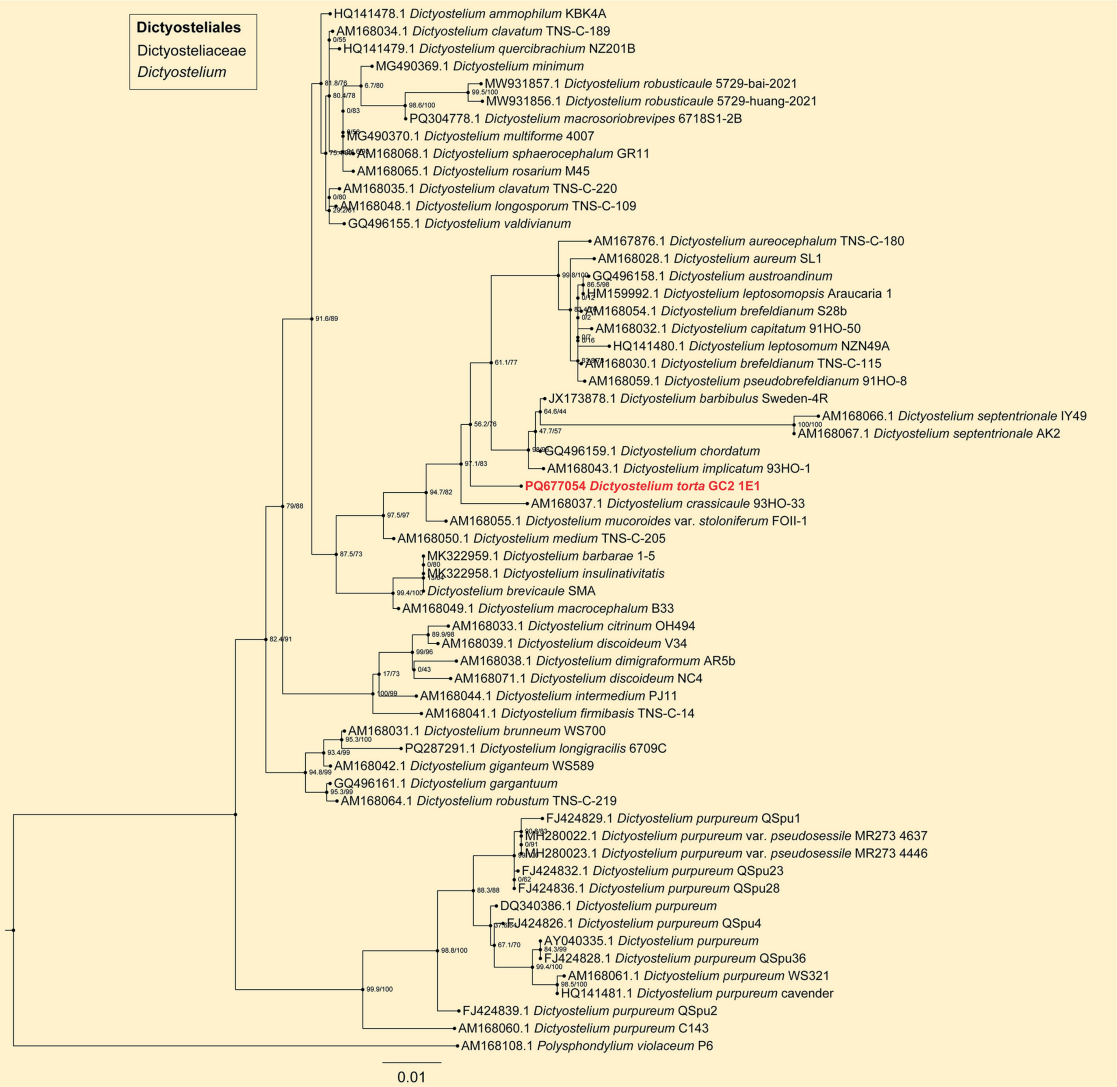
SSU phylogeny of *Dictyostelium* sequences in the order Dictyosteliales and the family Dictyosteliaceae. Numbers in parentheses are SH-aLRT support (%)/ultrafast bootstrap support (%). Scale bar represents substitutions per site. Newly generated sequences are indicated in bold.

**Fig 4:**
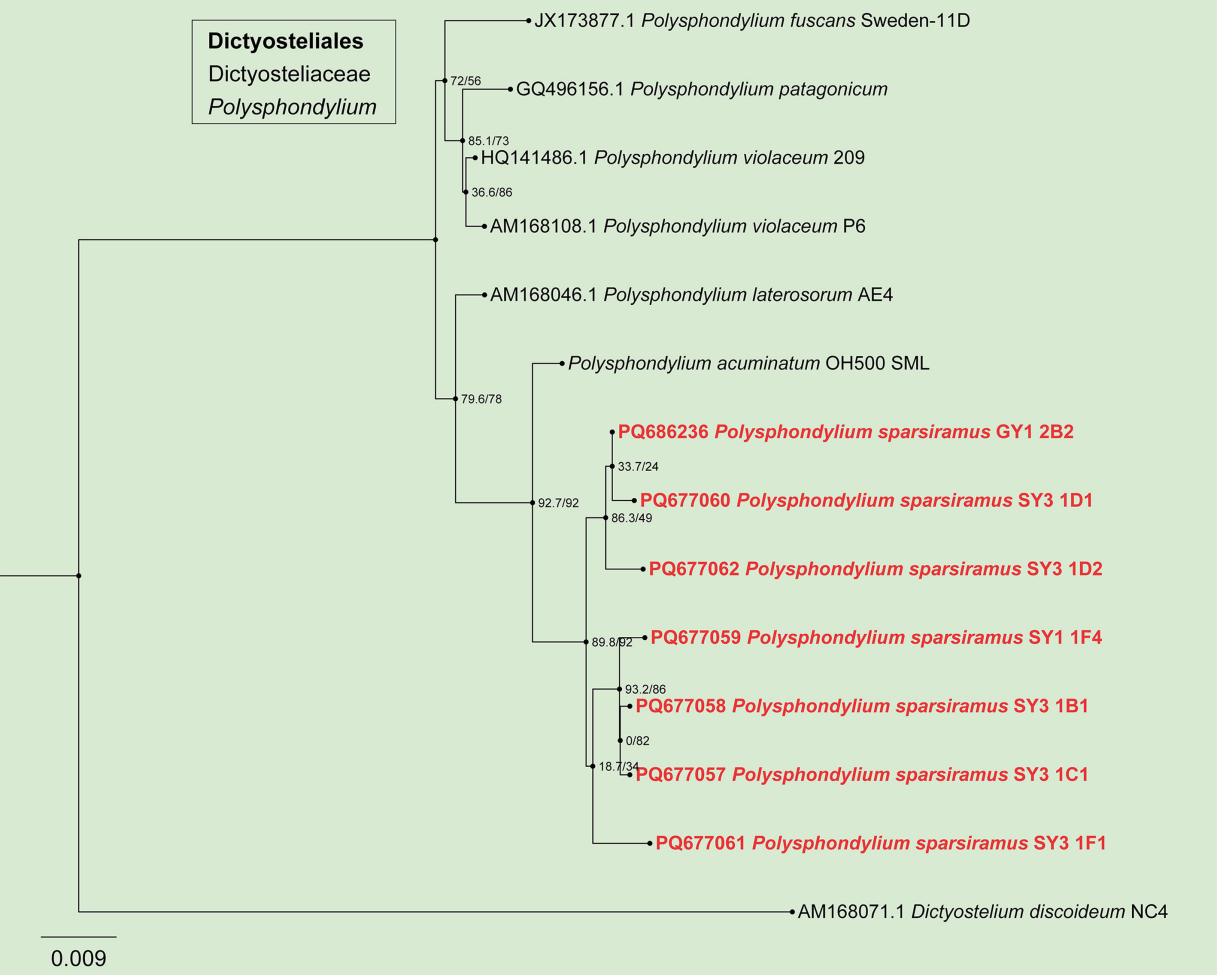
SSU phylogeny of *Polysphondylium* sequences in the order Dictyosteliales and the family Dictyosteliaceae. Numbers in parentheses are SH-aLRT support (%)/ultrafast bootstrap support (%). Scale bar represents substitutions per site. Newly generated sequences are indicated in bold.

**Fig 5:**
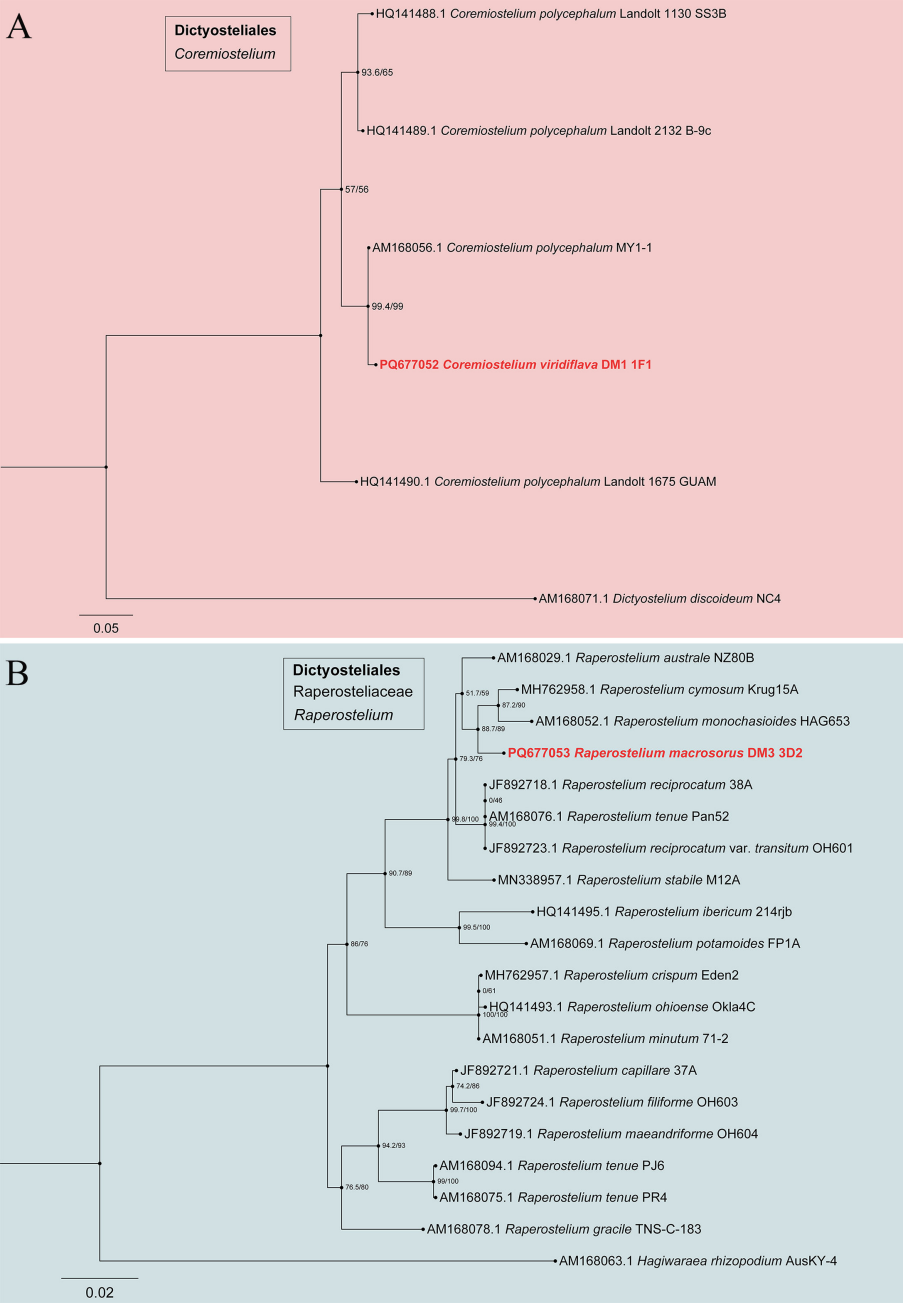
SSU phylogeny of *Coremiostelium* (**A**) and *Raperostelium* (**B**) sequences in the order Dictyosteliales. Numbers in parentheses are SH-aLRT support (%)/ultrafast bootstrap support (%). Scale bar represents substitutions per site. Newly generated sequences are indicated in bold.

**Fig 6:**
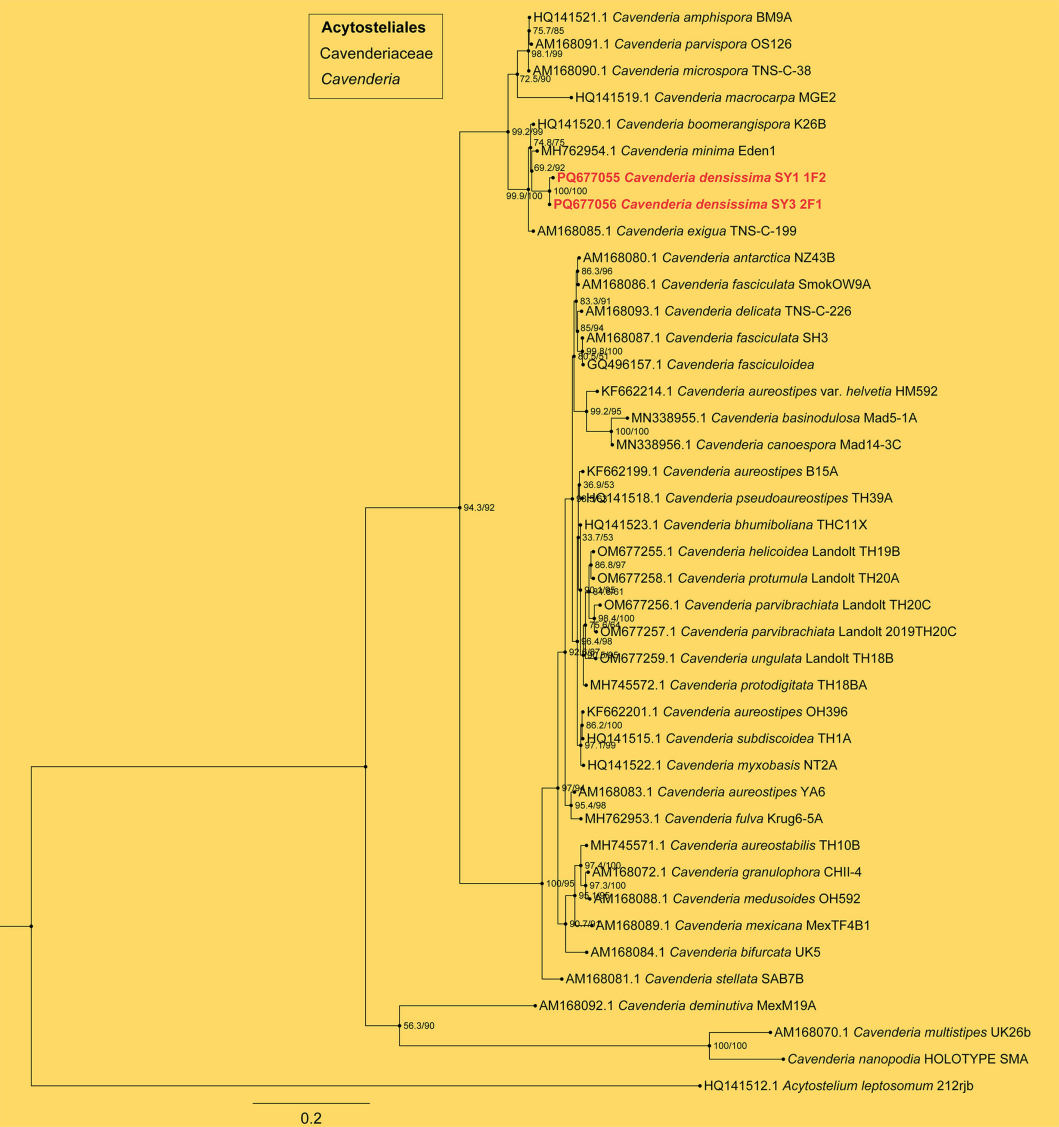
SSU phylogeny of *Cavenderia* sequences in the order Acytosteliales and the family Cavenderiaceae. Numbers in parentheses are SH-aLRT support (%)/ultrafast bootstrap support (%). Scale bar represents substitutions per site. Newly generated sequences are indicated in bold.

In the phylogenetic tree (Fig. 3 to 6), the SSU rDNA data revealed the five new species that are members of the orders Dictyosteliales and Acytosteliales, with scale bars representing substitutions per site. One strain (HMJAU MR445) belongs to *Dictyostelium*, with a SH-aLRT/ultrafast bootstrap support (%) value of 56.2/76 (Fig. 3). Eleven strains (HMJAU MR446–456) belong to *Polysphondylium*, where SH-aLRT/ultrafast bootstrap support (%) values were 33.7/24, 86.3/49, 89.8/92, 93.2/86, 0/82, and 18.7/34 (Fig. 4). One strain (HMJAU MR457) belongs to *Coremiostelium*, with a SH-aLRT/ultrafast bootstrap support (%) value of 99.4/99 (Fig. 5A). One strain (HMJAU MR458) belongs to *Raperostelium*, with a SH-aLRT/ultrafast bootstrap support (%) value = 88.7/89 (Fig. 5B). Six strains (HMJAU MR459-464) belong to *Cavenderia*, where the SH-aLRT/ultrafast bootstrap support (%) value was 100/100 (Fig. 6). The molecular data support our interpretations regarding these five newly identified species.

### Taxonomy

See Table 1 for morphological terms discussed below.

**Fig 7:**
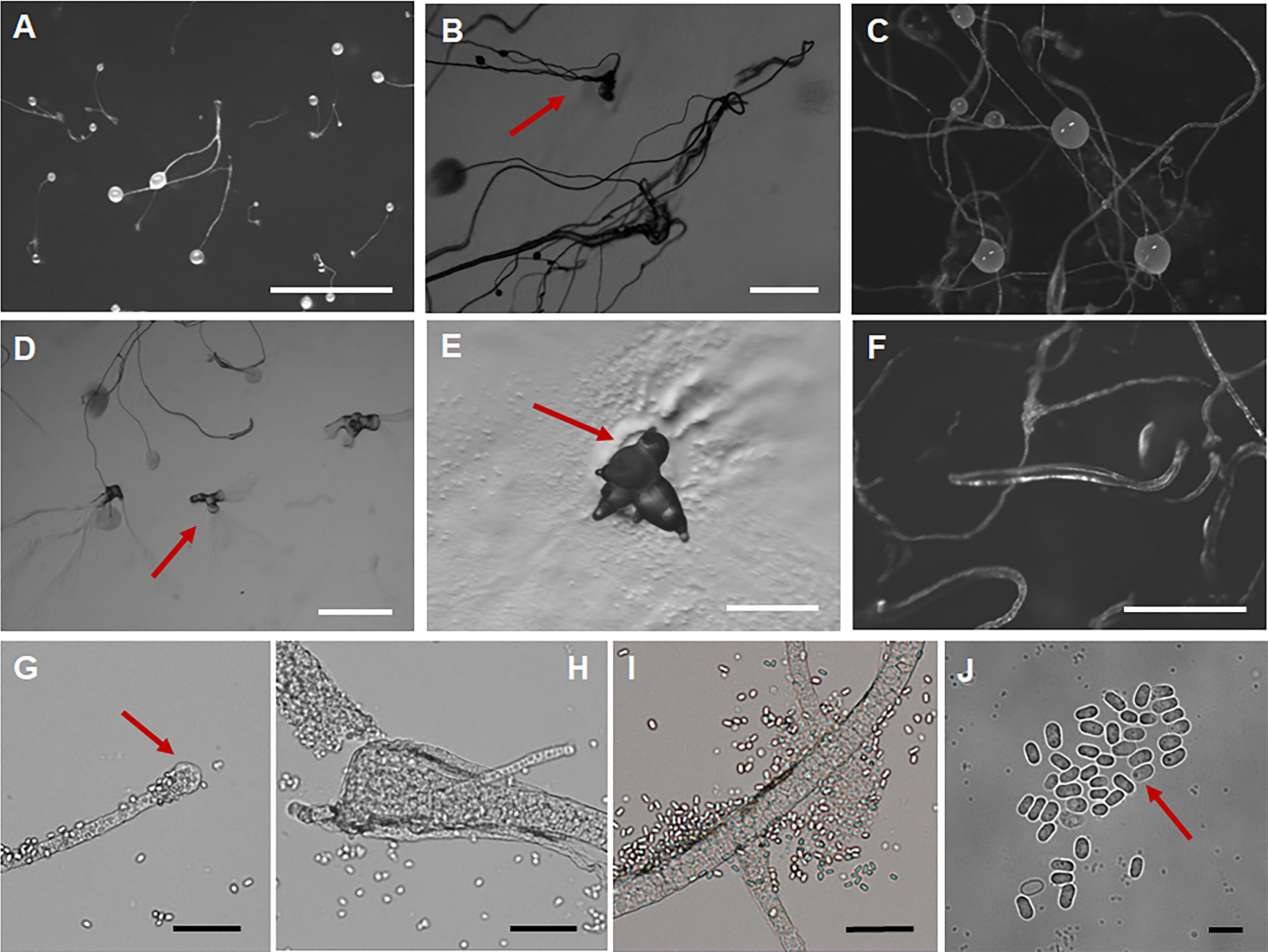
Morphological features of *Dictyostelium torta*. (**A, B**) Sorocarps. (**C**) Sori. (**D**) Aggregations. (**E**) Sorogen. (**F**) Pseudoplasmodia. (**G**) Sorophore tip. (**H**) Sorophore base. (**I**) Branches. (**J**) Spores. Scale bars: A, D = 2 mm; B = 1 mm; C, E, F = 0.5 mm; G–I = 50 µm; J = 10 µm. The red arrows refer to the key distinguishing characteristics.

*Dictyostelium torta* J.L. Jin, Z.J. Zhang, P. Liu, & Y. Li sp. nov. (Fig. 7).

Fungal Names accession number FN 572247.

When cultured at 23℃ on non-nutrient agar with *Escherichia coli*, **sorocarps** (Fig. 7A and B) solitary, clustered or gregarious, erect or inclined in early growth, often lodging in later growth, unbranched or with a small number of irregular branches, usually 1–4 branches, strongly phototropic, usually 0.611–4.650 mm in length. **Sorophores** colorless, sinuous, and often intertwined with each other. **Sorophore tips** (Fig. 7G) capitate, consisting of one or two rows of elongated cells, 5.058–14.962 (18.858) μm wide. **Sorophore bases** (Fig. 7H) clavate or conical, consisting of multiple rows of cells, 25.148–42.651 µm wide. **Sori** (Fig. 7C) globose or lemon-shaped, milky white translucent, 133–309 µm in diameter. **Spores** (Fig. 7J) usually oblong, sometimes subcircular, usually 1.27–1.93 times longer than broad, mostly 5.916–7.718 × 3.935–5.1 µm, without polar granules, scattered small particles. **Aggregations** (Fig. 7D) as mounds, with broad aggregations of myxamoebae. **Pseudoplasmodia** (Fig. 7F) migrating with the stalk.

Etymology. This name refers to the extremely twisted sorophores.

Holotype. HMJAU MR445 (Strain GC2 1E1) was isolated from 0–5 cm soil layer soil samples collected from a cabbage planting field in Huanzidong Village, Gongzhuling City, Changchun City, Jilin Province, China, on 25 August 2022 (Soil No. 7191, elevation 199.5 m, coordinates 43°30′23.76″N, 124°47′27.6″E).

GenBank accession number PQ677054.

Known distribution. Currently known only from China.

Commentary. Molecular phylogeny based on SSU rRNA supported the position of this species in the genus *Dictyostelium* (Fig. 3). It forms a clade together with *D. crassicanle*, but morphologically there are significant differences between the two species. The sorocarps of *D. crassicaule* (55) are solitary and non-phototropic, whereas those of *D. torta* are solitary or clustered and strong phototropic. *Dictyostelium torta* (0.611–4.650 mm) also is longer than *D. crassicanle* (0.2–2.2 mm). The aggregations of *D. crassicanle* are radiate, but in *D. torta,* they are mounds.

Morphologically, *D. torta* is characterized by sorophores that are often sinuous and intertwine. The only species of *Dictyostelium* with such a feature is *D. valdivianum* (56). However, the spores of *D. valdivianum* (4–7 × 2.5–3 µm) are smaller than those of *D. torta* (5.9–7.7 × 3.9–5.1 µm) and have distinct polar granules.

**Fig 8:**
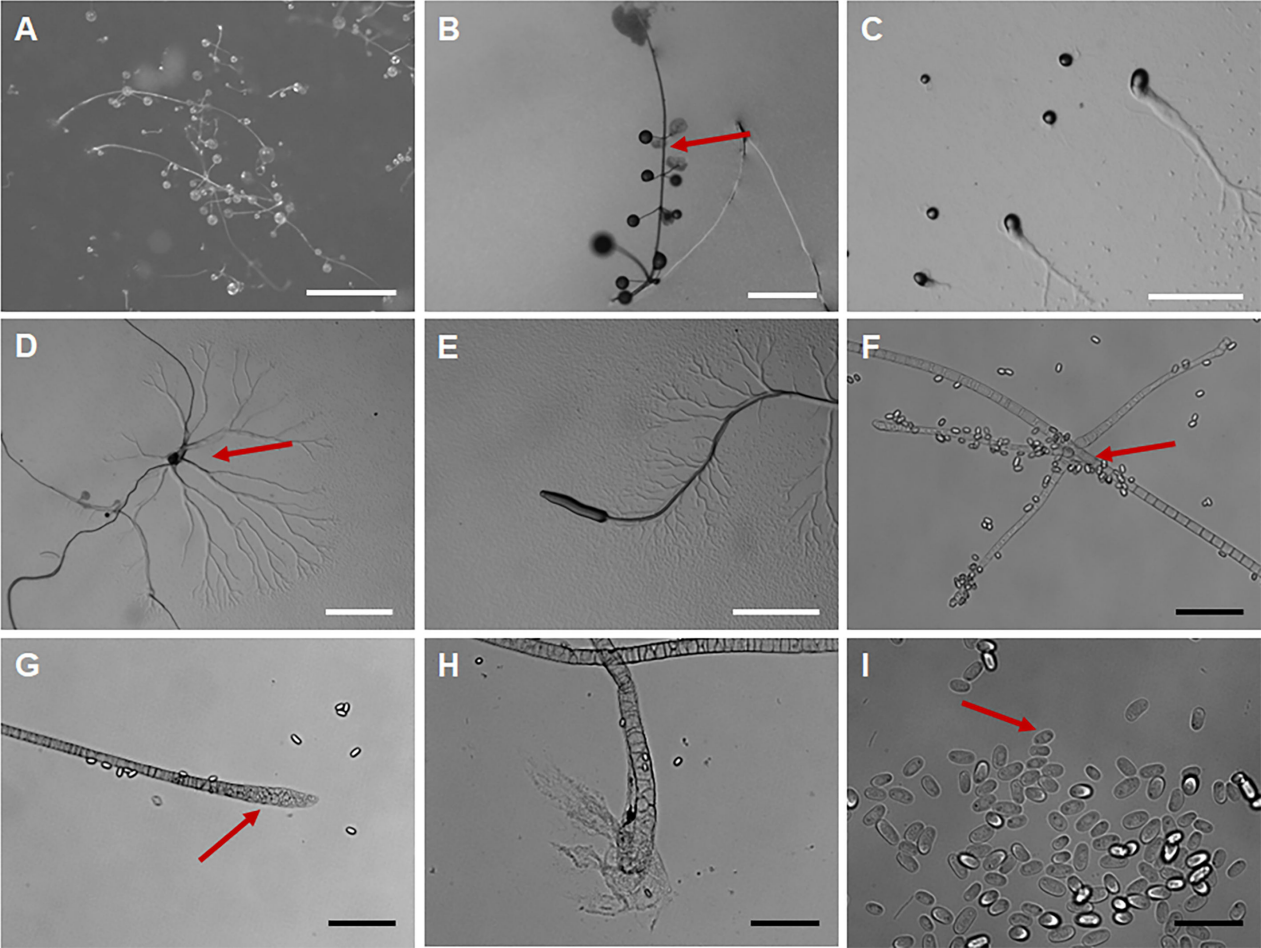
Morphological features of *Polysphondylium sparsiramus*. (**A, B**) Sorocarps. (**C, D**) Aggregations. (**E**) Pseudoplasmodia. (**F**) Branches. (**G**) Sorophore tip. (**H**) Sorophore base. (**I**) Spores. Scale bars: A, D, E = 1 mm; B, C = 0.5 mm; F–H = 50 µm; I = 20 µm. The red arrows refer to the key distinguishing characteristics.

*Polysphondylium sparsiramus* J.L. Jin, Z.J. Zhang, P. Liu, & Y. Li sp. nov. (Fig. 8).

Fungal Names accession number FN 572249.

When cultured at 23℃ on non-nutrient agar with *Escherichia coli*, **sorocarps** (Fig. 8A and B) solitary, erect, inclined or prone, whorled branches, but there are many small individuals without branches, phototropic. The length varies widely, usually between 0.5 and 8 mm, with a small number of individuals exceeding 1 cm in length. **Sorophores** lilac and slender except near the tip and the base consisting of 1–2 rows of cells. Tapering from the base to the tip, slightly enlarged near the top and consisting of 3 or more rows of small cells forming capitate or clavate **tips** (Fig. 8G), 4–15 µm wide. **Bases** (Fig. 8H) capitate or round, 8–18 µm, usually wrapped in mucus, 8–18 µm. **Whorls** commonly 1–8 (13). **Branches** (Fig. 8F) in a whorl commonly 1–4, occasional 5 branches. **Branches** of whorls 100–350 µm long, similar in morphology to the sorophore, nodes are regularly spaced, 0.5–0.9 mm, terminal segments usually 0.466–1.658 mm long. **Sori** fuchsia and globose, Terminal sori are 123–226 µm in diameter, sori of branches are 60–122 µm in diameter. **Spores** (Fig. 8I) lilac, elliptical, usually 1.68–2.23 times longer than broad, 5.79–8.43 × 3.49–4.18 µm, with or without polar granules (quantity 1–2). **Aggregations** (Fig. 8C and D) radial, sometimes a mound. **Pseudoplasmodia** (Fig. 8E) migrate with the stalk.

Etymology. This name refers to that the number of branches in each whorl is small, and they are sparse.

Holotype. HMJAU MR446–456 (Strains SY1 1B2, SY1 1C1, SY1 1E1, SY1 1F4, SY3 1B1, SY3 1B2, SY3 1C1, SY3 1D1, SY3 1D2, SY3 1F1, GY1 2B2) were isolated from 0–5 cm and 5–10 cm soil layer soil samples collected from a corn planting field in Xincheng Town, Ningjiang District, Songyuan City, and Dongwa community, Gongzhuling City, Changchun City, Jilin Province, China, on 26 August 2023 and 23 August 2022 (Strain GY1 2B2, Soil No. 7177, soil layer 5–10 cm, elevation 210.8 m, coordinates 43°31′32.16″N, 124°49′41.52″E; Strains SY1 1B2, SY1 1C1, SY1 1E1, SY1 1F4, soil no. 7808, soil layer 0–5 cm, elevation 142.2 m, coordinates 45°11′31.56″N, 124°51′5.76″E; Strains SY3 1B1, SY3 1B2, SY3 1C1, SY3 1D1, SY3 1D2, SY3 1F1, soil no. 7814, soil layer 0–5 cm, elevation 137.9 m, coordinates 45°11′25.8″N, 124°51′11.52″E).

GenBank accession numbers PQ677059, PQ677058, PQ677057, PQ677060, PQ677062, PQ677061, PQ686236.

Known distribution. Currently known only from China.

Commentary. *Polysphondylium sparsiramus* is characterized by solitary and purple sorocarps, a uniform distribution of cyclic whorled branches, and aggregation of the mucoroides-like type. The purple species of *Polysphondylium* include *P. acuminatum* (57), *P. fuscans* (58), and *P. violaceum* (23). The whorled branches of *P. sparsiramus* (1–8 whorls, branches in a whorl commonly 1–4, nodes are regularly spaced) are different from those of *P. acuminatum* (1–8 whorls, branches in a whorl 1–5, not regularly distributed), *P. fuscans* (1–3 whorls, branches in a whorl 2–6), *P. patagonicum* (56) (1–5 whorls, branches in a whorl 2–3), and *P. violaceum* (1–6 whorls, branches in a whorl 2–6). The aggregations of *P. sparsiramus* are the mucoroides-like type, unlike those of the other four species, which are the violaceum-like types. In addition, the length of the sorocarps of *P. sparsiramus* is very different from that of the other four species.

Molecular phylogeny based on SSU rRNA supported the position of this species in the genus *Polysphondylium* (Fig. 4). This species forms a clade with *P. acuminatum* and *P. laterosorum*. However, the sorus color of *P. laterosorum* (59) is bluish gray to light violet, and spores of *P. laterosorum* (elliptical, reniform or recurved, 6.0–13.0 µm × 2.5–4.0 µm) are clearly different from those of *P. sparsiramus* (elliptical, 5.79–8.43 × 3.49–4.18 µm).

**Fig 9:**
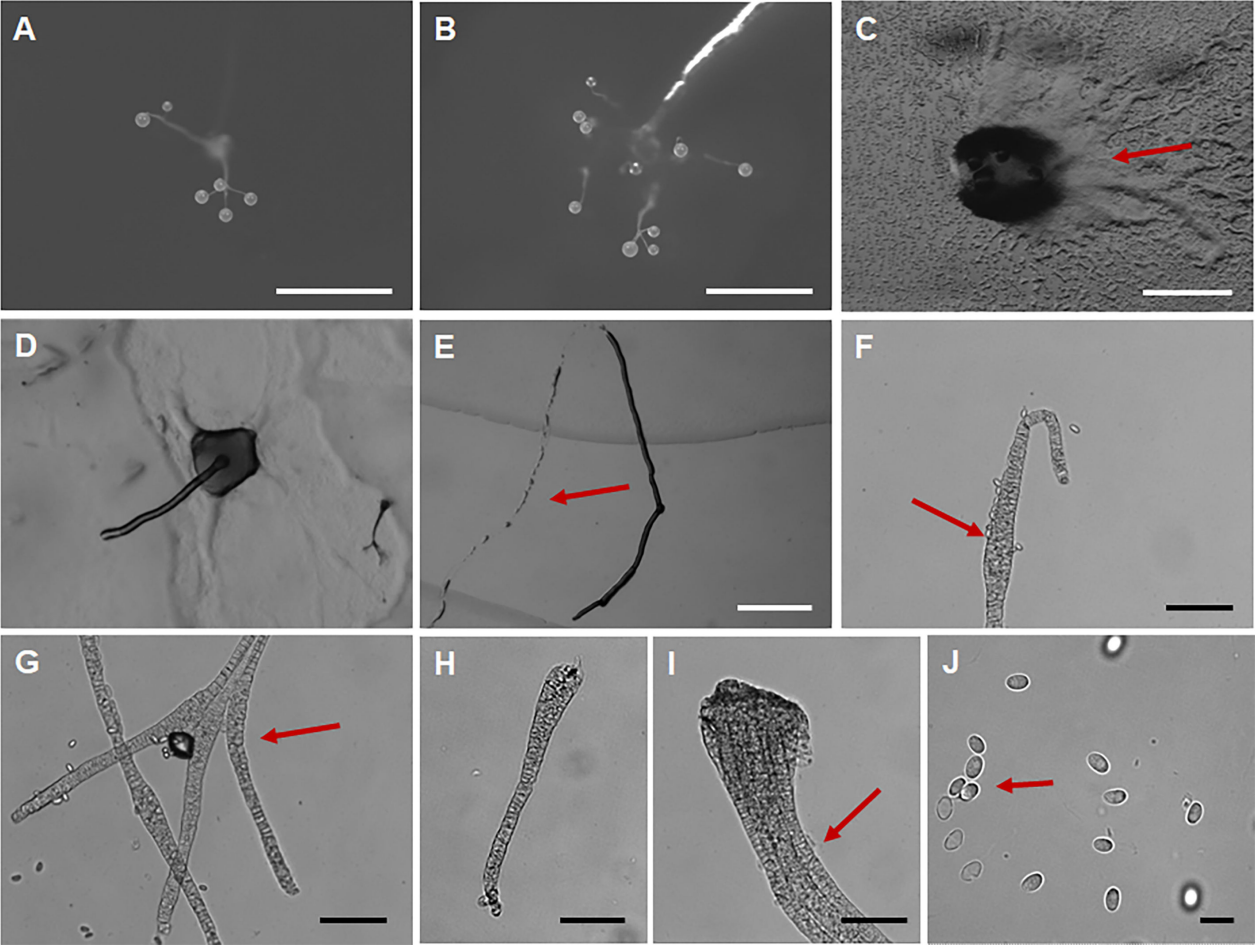
Morphological features of *Coremiostelium viridiflava*. (**A, B**) Sorocarps. (**C**) Aggregation. (**D**) Aggregation and Pseudoplasmodia. (**E**) Pseudoplasmodia. (**F, G**) Sorophore tips. (**H**) Sorophore. (**I**) Sorophore base. (**J**) Spores. Scale bars: A, B, E = 1 mm; C, D = 0.5 mm; F–I = 50 µm; J = 10 µm. The red arrows refer to the key distinguishing characteristics.

*Coremiostelium viridiflava* J.L. Jin, Z.J. Zhang, P. Liu, & Y. Li sp. nov. (Fig. 9).

Fungal Names accession number FN 572245.

When cultured at 23 ℃ on non-nutrient agar with *Escherichia coli*, **sorocarps** (Fig. 9A and B) solitary or coremium-like, when sorocarps clustered to form coremiform fructifications with the number of sorocarps ranging from 2–4, and a small number can reach 5. **Sorocarps** erect, short, tough, unbranched, non-lodging, non-phototropic, 0.402–0.906 mm high. **Sorophores** (Fig. 9H) are chartreuse under the microscope, slender, consisting of one to three tiers of cells, whole sorophore is relatively uniform, when the sorocarps are coremium-like there is an obvious thickening in different individual dispersions, and thickening in the middle-upper parts of solitary individual sorophores. **Sorophore tips** (Fig. 9F and G) clavate or capitate (a single round cell), clean without spore and mucous adhesion, usually composed of two rows of cells, 7.935–14.637 µm wide. **Bases** (Fig. 9I) clavate, composed of 1–2 rows of cells, 7.962–14.818 µm wide. **Sori** globose, initially milky and translucent, becoming grayish white as moisture decreases, 101–205 µm in diameter. **Spores** (Fig. 9J) elliptical, chartreuse, usually 1.30–1.81 times longer than broad, surface particles, 5.895–7.454 × 3.711–5.249 µm, granules often evident but not consistently polar. **Aggregations** (Fig. 9C and D) as mounds, sometimes with a few stout amoeba collection flows. **Pseudoplasmodia** (Fig. 9D and E) often migrate and can migrate for a long distance, with obvious traces of migration, and the residual pseudoplasmodia can sometimes form smaller **sorogens** during migration.

Etymology. This name refers to the chartreuse sorophores and spores.

Holotype. HMJAU MR457 (Strain DM1 1F1) was isolated from 0–5 cm soil samples collected from a soybean planting field in Dongliao County, Liaoyuan City, Jilin Province, China, on 22 August 2023 (Soil No. 7709, elevation 266.8 m, coordinates 42°55′19.92″N, 124°59′34.8″E).

GenBank accession number PQ677052.

Known distribution. Currently known only from China.

Commentary. *Coremiostelium viridiflava* belongs to the *Coremiostelium* clade in a SSU rDNA phylogeny (Fig. 5A). Species of *Coremiostelium* are rare with only *Co. polycephalum* previously known. Compared with the growth temperature of *Co. polycephalum* (23) (28–30℃), the culture temperature of this species is lower, and the complete life history can be carried out at 23℃. *Coremiostelium polycephalum* has a large number of coremium-like growth individuals, even up to 9–10, while the coremium-like growth individuals of this strain are usually 2–4, sometimes up to 5. The aggregations of *Co. polycephalum* are radiate, whereas the aggregations of this species are mound-like. The sorus diameter of *Co. polycephalum* (40–90 µm) is smaller than *Co. viridiflava* (100–200 µm). Moreover, the spores of *Co. viridiflava* (5.895–7.454 × 3.711–5.249 µm) are larger than *Co. polycephalum* (6.0–7.5 × 3.0–3.5 µm).

**Fig 10:**
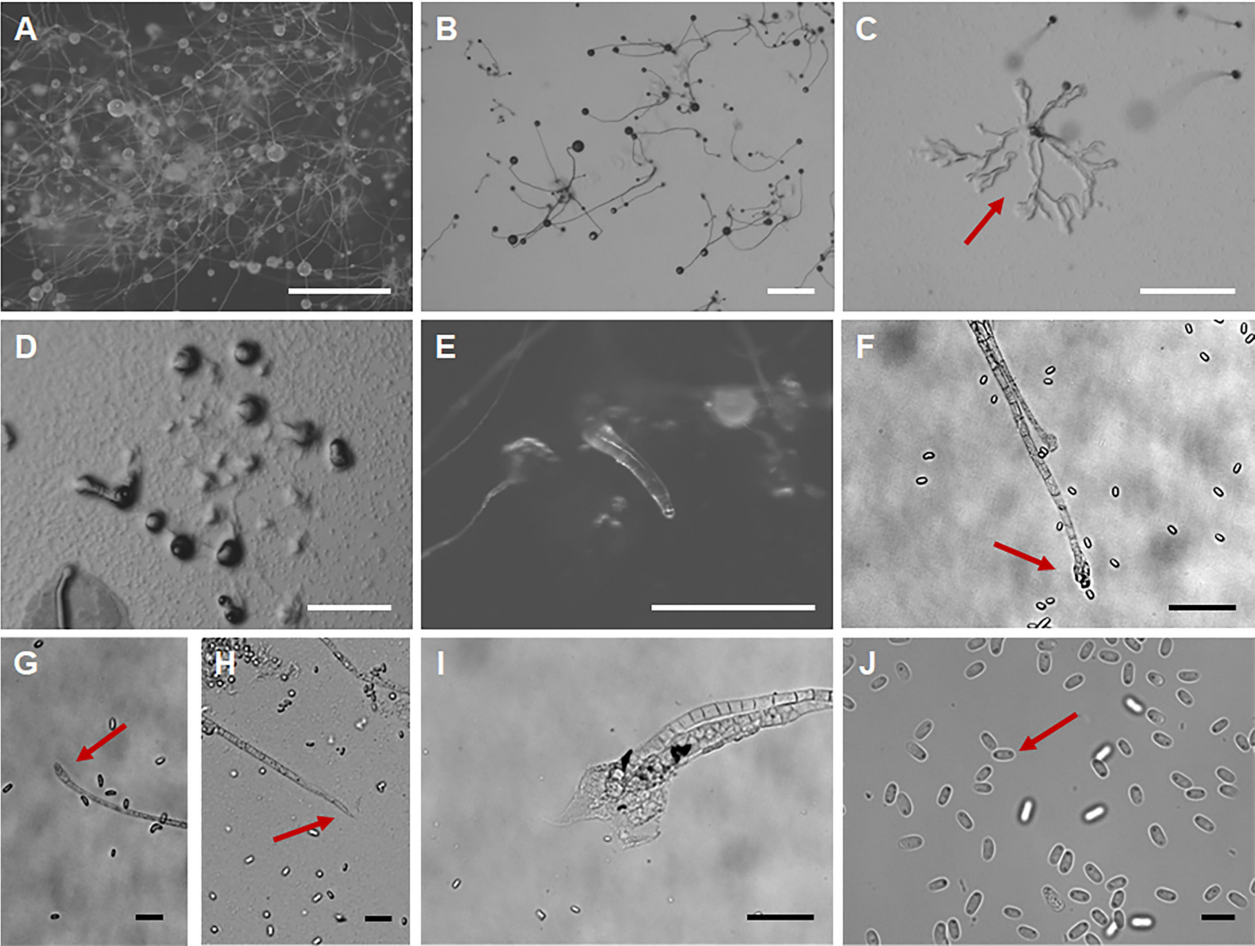
Morphological features of *Raperostelium macrosorus*. (**A, B**) Sorocarps. (**C, D**) Aggregations. (**E**) Pseudoplasmodia. (F–H) Sorophore tips. (**I**) Sorophore base. (**J**) Spores. Scale bars: A = 1 mm; B–E = 0.5 mm; F, I = 50 µm; G, H = 20 µm; J = 10 µm. The red arrows refer to the key distinguishing characteristics.

*Raperostelium macrosorus* J.L. Jin, Z.J. Zhang, P. Liu, & Y. Li sp. nov. (Fig. 10).

Fungal Names accession number FN 572246.

When cultured at 23℃ on non-nutrient agar with *Escherichia coli*, **sorocarps** (Fig. 10A and B) growth types are diverse, solitary, clustered, or gregarious. **Sorocarps** erect or inclined, unbranched, sometimes there are a few individuals with a few branches, usually 1–2 branches, delicate and prone to lodging, weakly phototropic, 0.281–1.064 mm high. **Sorophore tips** (Fig. 10F–H) clavate, capitate, or acuminate, usually consisting of a single row of elongated cells, 2.467–4.284 µm wide. **Sorophore bases** (Fig. 10I) clavate or conical, usually consisting of 1–2 rows of cells, 8.734–16.386 µm wide. **Sori** globose, milky white translucent, 142–310 µm in diameter. **Spores** (Fig. 10J) elliptical to long elliptical, usually 1.91–2.41 times longer than broad, surface particles, 6.307–8.549 × 2.620–3.823 µm (mostly 6.5–7.5 × 3–3.8 µm), with polar granules, usually 1–3 in number. **Aggregations** (Fig. 10C and D) are violaceum-like. Atypical radiate aggregations, forming multiple secondary centers. **Pseudoplasmodia** (Fig. 10E) migrating with stalks.

Etymology. This name refers to the large sorus relative to the delicate sorophores.

Holotype. HMJAU MR458 (Strain DM3 3D2) was isolated from a 10–15 cm soil layer soil sample collected from a soybean planting field in Dongliao County, Liaoyuan City, Jilin Province, China, on 22 August 2023 (Soil No. 7717, elevation 268.5 m, coordinates 42°55′17.4″N, 124°59′36.24″E).

GenBank accession number PQ677053.

Known distribution. Currently known only from China.

Commentary. *R. macrosorus* belongs to the *Raperostelium* clade in a SSU rDNA phylogeny (Fig. 5B). Morphologically, *R. macrosorus* is characterized by unbranched sorocarps and sorophore tips clavate or acuminate. Species in the genus *Raperostelium* usually have many branches and piliform tips, but this species has few branches, sorophore tips clavate, capitate, or acuminate. Similar species are *R. gracile* (60) and *R. australe* (61). However, *R. macrosorus* growth habits are mainly clustered, while *R. garile* is mainly solitary. *Raperostelium macrosorus* (142–310 µm diameter) has larger sori and spores than *R. gracile* (20–180 µm diameter) and *R. australe* (40–180 µm diameter). The sorophore of *R. macrosorus* (tips 2–5 µm, bases 8–17 µm) is slimmer than that of *R. australe* (tips 3–15 µm, bases 15–40 µm). Moreover, the morphology of the sorophore tip of *R. macrosorus* (clavate, capitate, or acuminate) is different from those of *R. gracile* (blunt, sometimes hair-pointed) and *R. australe* (terminal segment flexuous or with capitate).

**Fig 11:**
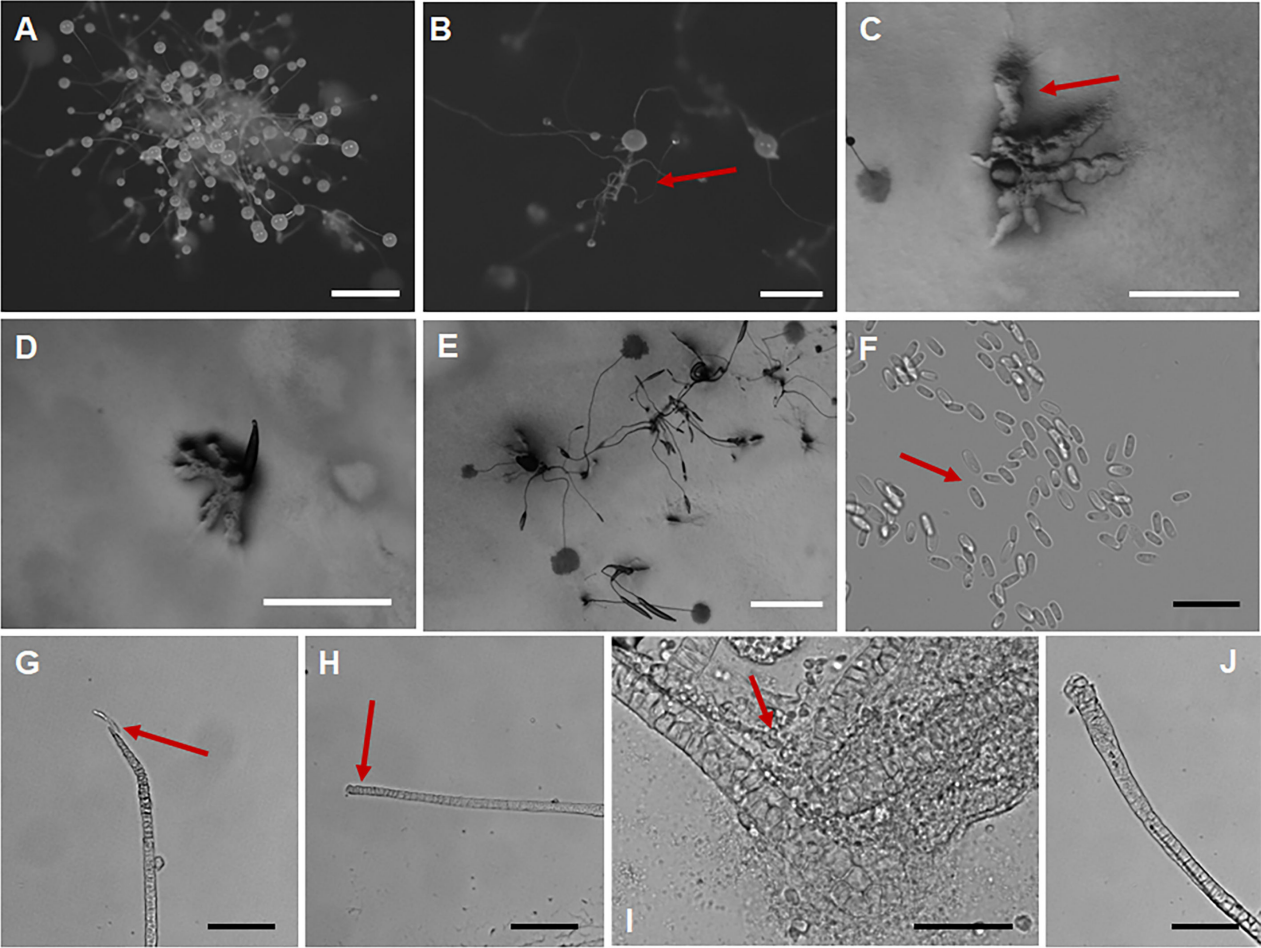
Morphological features of *Cavenderia densissima*. (**A, B**) Sorocarps. (**C, D**) Aggregations. (**E**) Pseudoplasmodia. (**F**) Spores. (**G, H**) Sorophore tips. (**I, J**) Sorophore bases. Scale bars: A, E = 1 mm; B–D = 0.5 mm; F = 20 µm; G–J = 50 µm. The red arrows refer to the key distinguishing characteristics.

*Cavenderia densissima* J.L. Jin, Z.J. Zhang, P. Liu, & Y. Li sp. nov. (Fig. 11).

Fungal Names accession number FN 572248.

When cultured at 23℃ on non-nutrient agar with *Escherichia coli*, **sorocarps** (Fig. 11A and B) generally densely clustered, sometimes solitary, hyaline-white, erect, inclined or semierect, delicate and prone to lodging, unbranching or having an indefinite number of irregular branches, branches usually 0.293–1.174 mm long, non-phototropic, usually 0.911–4.822 mm high. **Sorephores** colorless, tapering from base to tip. Except for the base, the entire body consists of a single row of cells. **Sorophore tips** (Fig. 11G and H) capitate or clavate, 4.209–8.567 µm wide. **Sorophore bases** (Fig. 11I and J) clavate or obtuse, consisting of two to three rows of cells, no mucus adhesion, 11.589–27.409 µm wide. **Sori** globose, milky white translucent, 104–246 µm in diameter. **Spores** (Fig. 11F) slender, oblong, usually 2.19–2.74 times longer than broad, 5.049–7.765 × 2.207–3.065 µm, with two distinct polar granules distributed at both ends of the spore. **Aggregations** (Fig. 11C and D) were irregular. At first, with short cell streams forming mound-like aggregations and then continued to aggregate around the mounds, forming toe-like or irregular aggregations. **Pseudoplasmodia** (Fig. 11E) does not migrate.

Etymology. This name refers to the densely clustered sorocarps.

Holotype. HMJAU MR459–464 (Strains SY1 1D2, SY1 1F2, SY2 2B2, SY2 2B3, SY3 1F4, SY3 2F1) were isolated from 0–5 cm and 5–10 cm soil layer soil samples collected from a corn planting field in Xincheng Town, Ningjiang District, Songyuan City, Jilin Province, China, on 26 August 2023 (Strains SY1 1D2, SY1 1F2, soil no. 7808, soil layer 0–5 cm, elevation 142.2 m, coordinates 45°11′31.56″N, 124°51′5.76″E; Strains SY2 2B2, SY2 2B3, soil no. 7812, soil layer 5–10 cm, elevation 145.6 m, coordinates 45°11′43.08″N, 124°50′53.16″E; Strains SY3 1F4, SY3 2F1, soil no. 7814, soil layer 0–5 cm and 7815, soil layer 5–10 cm, elevation 137.9 m, coordinates 45°11′25.8″N, 124°51′11.52″E).

GenBank accession number PQ677055, PQ677056.

Known distribution. Currently known only from China.

Commentary. This species is distinguished by its habit of having toe-like aggregations and dense clusters. A molecular phylogeny based on SSU rRNA supported the position of this species in the genus *Cavenderia* (Fig. 6). It forms a clade together with *C. minima*, *C. boomerangispora,* and *C. exigua*. However, there are significant differences in their morphology. The sorephores of *C. densissima* (0.9–4.8 mm) are longer than those of *C. minima* (62) (0.2–2 mm), *C. boomerangispora* (63) (0.5–1.0 mm), and *C. exigua* (60) (0.2–2.8 mm). The spores of *C. densissima* (5.049–7.765 × 2.207–3.065 µm) are longer than those of *C. exigua* (4.4–6.1 × 2.4–3.1 µm). The spores of *C. boomerangispora* are often curved and boomerang-shaped and can be distinguished from the oblong spores of *C. densissima*. The sorophore of *C. densissima* (no mucus adhesion, tips capitate or clavate, bases 11.589–27.409 µm wide) is different from that of *C. boomerangispora* (tips oblong to piliform, bases often with the upper part interrupted by dense slime and undifferentiated lateral cells, 5–7 µm in diameter) and *C. minima* (with a dense hyaline sheath, tips capitate flexuous, bases within a disk-like mass of conical mucilage).

## DISCUSSION

### Microbial contributions to agricultural ecosystem sustainability

The agricultural ecosystem (Fig. 1) is a multifaceted entity, encompassing intricate biological communities that span beyond plants and animals to encompass a vast and diverse array of microorganisms. These microorganisms, including archaea, bacteria, fungi, and protists, play indispensable roles in maintaining soil health, orchestrating nutrient cycling, and ensuring plant vitality (64, 65). In the context of intensifying land use, recognizing the complexity of soil multitrophic networks emerges as a pivotal factor for augmenting agricultural ecosystem productivity and sustainability. By developing a deeper understanding of these microbial interactions, we can better harness their potential to address the myriad challenges faced by modern agriculture (66). Notably, plant-associated microorganisms represent an untapped reserve of solutions, offering promising avenues for enhancing agricultural resilience and sustainability (1, 67, 68).

### Discovery of novel dictyostelid species in agricultural ecosystems

Our investigation of agricultural ecosystems, specifically corn, cabbage, and soybean fields in black soil regions of Jilin Province, has yielded noteworthy discoveries. A total of five novel dictyostelid species, comprising 20 strains, were identified through meticulous morphological and molecular phylogenetic analyses (Fig. 2 to 11). These newly recognized taxa belong to three distinct families (28)—the Dictyosteliaceae, Raperosteliaceae, and Cavenderiaceae. The recognition of *Coremiostelium viridiflava* as a novel species, despite limited SSU rDNA divergence from *Co. polycephalum* (Fig. 5A), underscores the necessity of using integrative taxonomy in dictyostelid systematics. While genetic markers like SSU rRNA are highly conserved (28, 69), morphological traits (Fig. 9) such as sorus size, spore size, aggregation patterns, and sorocarp organization remain pivotal for species delineation (23, 25). The ecological and functional implications of these morphological differences warrant further investigation.

Traditionally, dictyostelids have been mostly isolated from forest habitats (32, 35, 40, 70, 71). However, our findings expand their known distribution, as only 14 dictyostelid species have previously been reported from agricultural environments. These are *Dictyostelium giganteum*, *D. sphaerocephalum*, *D. irregularis*, *D. arabicum*, *D. macrocephalum*, *D. magnum*, *D. purpureum*, *D. mucoroides*, *Heterostelium multicystogenum*, *H. pallidum*, *Hagiwaraea lavandula*, *Polysphondylium violaceum*, *Coremiostelium polycephalum*, and *Cavenderia aureostipes* (36–41) (Table S1). Among these, only four species—*D. giganteum*, *D. sphaerocephalum*, *D. mucoroides*, and *Cavenderia aureostipes*—have been documented as occurring in Chinese agricultural ecosystems (40, 41). This discovery underscores the potential richness of microbial diversity in agricultural soils and highlights the importance of continued exploration to uncover additional microbial species that may contribute to agricultural sustainability.

### Advancements in dictyostelid research through SSU sequencing

The availability of DNA sequencing data for dictyostelids has witnessed a steady surge in recent years (33, 62, 72–74). Currently, there are 168 recognized species of dictyostelids, with sequence information on the SSU rRNA gene available for 212 strains (Table S3). This wealth of sequence data has significantly contributed to the identification of newly recognized species and facilitated their accurate and rapid identification (27, 28, 75). While the internal transcribed spacer (ITS) region has limitations in species identification within dictyostelids, the utilization of SSU rRNA sequence data has greatly enhanced the precision of species identification and the elucidation of phylogenetic relationships among dictyostelids. In this study, we generated 12 sequence data sets for the SSU gene region, which will undoubtedly propel further research into dictyostelids. These data sets will serve as invaluable resources for the scientific community, enabling more comprehensive and nuanced studies of dictyostelid diversity, ecology, and potential applications in agriculture and beyond.

### Limitations to the current study

Based on our previous studies of dictyostelids in agricultural ecosystems, the five species identified in this study can be expected to be characterized by high biological activity. However, one must also consider the limitations of this type of research. First, limitations arise from the cultivability of microorganisms. Specifically, DNA recovery from some eukaryotic cell morphologies, especially those of dictyostelid spores, is less efficient compared to others. This technical challenge may hinder the molecular identification and validation of certain strains isolated from environmental samples. Without reliable DNA-based confirmation, it becomes difficult to establish pure cultures or ensure the taxonomic integrity of strains during long-term laboratory maintenance. Consequently, this limits their availability for functional studies or agricultural applications (e.g., biofertilizer development), as uncharacterized or misidentified strains are less likely to be prioritized for applied research.

Second, given the focus on corn, cabbage, and soybean soil samples from specific locations in Jilin Province, the sampling done in the present study may not be comprehensive enough to fully capture the diversity of dictyostelids in the broader agroecosystem. For instance, rare or region-specific species might remain undetected, thus potentially overlooking taxa with unique ecological roles. Future research should involve more extensive sampling across different crop types, soil types, and geographical regions within the black soil region to gain a more comprehensive understanding of dictyostelid diversity.

Finally, improvements in sequencing technology (e.g., long-read sequencing for higher resolution) and bioinformatics tools (e.g., machine-learning-based taxonomic classification) are necessary to overcome the aforementioned challenges of low DNA recovery and incomplete sampling. Such advancements would enhance the detection of cryptic species, refine phylogenetic analyses, and ultimately support the discovery of novel microbial resources with potential agricultural value.

## Data Availability

Data will be made available on request.

## SUPPLEMENTAL MATERIAL

Supplemental tables aem.00025-25-s0001.docx Tables S1 to S3.

ASM does not own the copyrights to Supplemental Material that may be linked to, or accessed through, an article. The authors have granted ASM a non-exclusive, world-wide license to publish the Supplemental Material files. Please contact the corresponding author directly for reuse.
