# Supplementary material for: The overlooked amoebae of an agroecosystem of black soil land in China: five new species of dictyostelids
Source: Appl Environ Microbiol. 2025 Jun 10;91(7):e00025-25. doi: 10.1128/aem.00025-25 (PMC12285224; doi:10.1128/aem.00025-25)
Supplement: Supplemental tables — Tables S1 to S3. [file aem.00025-25-s0001.docx]

Supplemental Table 1. Summary information on the dictyostelids that have been reported from agricultural soils.

| **Species** | **Agricultural environment** | **Distribution** | **References** |  |
| --- | --- | --- | --- | --- |
| *Dictyostelium giganteum* | Agricultural soils (tilled and untilled soil) | Fayetteville in northwestern Arkansas, United States | (36) |  |
| *Dictyostelium sphaerocephalum* |  |  |  |  |
| *Dictyostelium irregularis* | Decaying ear of corn | Hawaii, United States | (37) |  |
|  |  |  |  |  |
| **Heterostelium multicystogenum* | Soils of cultivated fields | Sierra Leone, West Africa | (38) |  |
|  |  |  |  |  |
| *Dictyostelium arabicum* | Soils of a tomato field in a highland region | Dhufar Province, Oman | (39) |  |
| **Hagiwaraea lavandula* |  |  |  |  |
| *Dictyostelium macrocephalum* |  |  |  |  |
| *Dictyostelium magnum* |  |  |  |  |
| *Dictyostelium purpureum* |  |  |  |  |
| **Polysphondylium violaceum* |  |  |  |  |
| **Heterostelium pallidum* complex |  |  |  |  |
| *Dictyostelium macrocephalum* | Soils of a grapefruit orchard in a highland region |  |  |  |
| *Dictyostelium magnum* |  |  |  |  |
| **Coremiostelium polycephalum* |  |  |  |  |
| *Dictyostelium purpureum* |  |  |  |  |
| **Polysphondylium violaceum* |  |  |  |  |
| **Heterostelium pallidum* complex |  |  |  |  |
| *Dictyostelium giganteum* | Farmland soils | Gansu Province, China | (40) |  |
| *Dictyostelium sphaerocephalum* |  |  |  |  |
| *Dictyostelium mucoroides* |  |  |  |  |
| *Cavenderia aureostipes* | Farmland soils (scallion field) | Jilin Province, China | (41) |  |
|  |  |  |  |  |

*indicates the name in the new classification system (28).

**References**

28. Sheikh S, Thulin M, Cavender JC, Escalante R, Kawakami S–i, Lado C, Landolt JC, Nanjundiah V, Queller DC, Strassmann JE, Spiegel FW, Stephenson SL, Vadell EM, Baldauf SL. 2018. A new classification of the dictyostelids. Protist 169:1–28. https://doi.org/10.1016/j.protis.2017.11.001

36. Stephenson SL, Rajguru SN. 2010. Dictyostelid cellular slime moulds in agricultural soils. Mycosphere 1:333–336.

37. Nelson N, Olive LS, Stoianovitch C. 1967. A new species of *Dictyostelium* from Hawaii. American Journal of Botany 54:354–358. https://doi.org/10.2307/2440763

38. Kawakami SI, Hagiwara H. 2008. *Polysphondylium multicystogenum* sp nov., a new dictyostelid species from Sierra Leone, West Africa. Mycologia 100:347–351. https://doi.org/10.3852/mycologia.100.2.347

39. Hagiwara H. 1991. A new species and some new records of dictyostelid cellular slime molds from Oman. Bulletin of the National Science Museum, Tokyo, Series B 17:109–121.

40. Zhang Z, Yang Y, Zhao J, Li Y, Stephenson SL, Qiu J, Liu P. 2023. Environmental factors influencing the diversity and distribution of dictyostelid cellular slime molds in forest and farmland soils of western China. Microbiology Spectrum 11:e01732–23. https://doi.org/10.1128/spectrum.01732–23

41. Zhang ZJ, He L, Sun YQ, Li Z, Yang YK, Zhai C, Stephenson SL, Xie XR, Li Y, Liu P. 2024. New species and records expand the checklist of cellular slime molds (dictyostelids) in Jilin Province, China. Journal of Fungi 10:1–23. <https://doi.org/10.3390/jof10120834>

Supplemental Table 2. Summary information on the soil samples processed for the isolation of

dictyostelids in the present study.

| **Soil nos.** | **Localities** | **Crops** | **Soil type** | **Soil depth (cm)** | **Coordinates** | **Elevation  (m)** | **Weather** | **Humidity** | **Dictyostelid  species** | **Strain  no.** |
| --- | --- | --- | --- | --- | --- | --- | --- | --- | --- | --- |
| 7177 | Dongwa community, Gongzhuling City,  Changchun City (L1) | Corn | Mollisol | 5–10 | 43°31'32.16"N, 124°49'41.52"E | 210.8 | Sunny,  12–23°C | 0.6 | ****Polysphondylium  sparsiramus*** (1) | GY1 2B2 |
| 7191 | Huanzidong Village,  Gongzhuling City,  Changchun City (L2) | Cabbage | Mollisol | 0–5 | 43°30'23.76"N,  124°47'27.6"E | 199.5 | Partly cloudy,  14–24°C | 0.8 | ****Dictyostelium  torta*** (1) | GC2 1E1 |
| 7709 | Dongliao County,  Liaoyuan City (L3) | Soybean | Mollisol | 0–5 | 42°55'19.92"N, 124°59'34.8"E | 266.8 | Overcast to  cloudy,  14–27°C | 0.8 | ****Coremiostelium  viridiflava*** (1) | DM1 1F1 |
| 7717 |  |  |  | 10–15 | 42°55'17.4"N, 124°59'36.24"E | 268.5 |  | 0.7 | ****Raperostelium  macrosorus*** (1) | DM3 3D2 |
| 7808 | Xincheng Town, Ningjiang District, Songyuan City (L4) | Corn | Chernozem | 0–5 | 45°11'31.56"N, 124°51'5.76"E | 142.2 | Sunny,  15–25°C | 0.4 | ********Cavenderia  densissima*** (2) | SY1 1D2,  SY1 1F2 |
|  |  |  |  |  |  |  |  |  | ****Polysphondylium  sparsiramus*** (4) | SY1 1B2, SY1 1C1, SY1 1E1, SY1 1F4 |
| 7812 |  |  |  | 5–10 | 45°11'43.08"N, 124°50'53.16"E | 145.6 |  | 0.5 | ****Cavenderia  densissima*** (2) | SY2 2B2, SY2 2B3 |
| 7814 |  |  |  | 0–5 | 45°11'25.8"N, 124°51'11.52"E | 137.9 |  |  | ****Cavenderia  densissima*** (1) | SY3 1F4 |
|  |  |  |  |  |  |  |  |  | ****Polysphondylium  sparsiramus*** (6) | SY3 1B1, SY3 1B2, SY3 1C1, SY3 1D1, SY3 1D2, SY3 1F1 |
| 7815 |  |  |  | 5–10 |  |  |  |  | ****Cavenderia  densissima*** (1) | SY3 2F1 |

The numbers (1), (2), (4), (6) indicate the numbers of species strains obtained from the soil samples (n). *refers to a species new to science.

Supplemental Table 3. A list of all the strains included in the phylogenetic analysis.

| **Species name** | **Strain number** | **SSU accession numbers** | **Species name** | **Strain number** | **SSU accession numbers** |  |
| --- | --- | --- | --- | --- | --- | --- |
|  |  |  |  |  |  |  |
| *Cavenderia amphispora* | BM9A | HQ141521.1 | *H. lavandula* | B15 | AM168047.1 |  |
| *C. antarctica* | NZ43B | AM168080.1 | *H. radiculata* | ML5A | HQ141494.1 |  |
| *C. aureostabilis* | TH10B | MH745571.1 | *H. rhizopodium* | AusKY-4 | AM168063.1 |  |
| *C. aureostipes* | YA6 | AM168083.1 | *H. tenebrica* | Ong2 | MH762956.1 |  |
| *C. aureostipes* | B15A | KF662199.1 | *H. vinaceofusca* | CC4 | AM168062.1 |  |
| *C. aureostipes* | OH396 | KF662201.1 | *Raperostelium  australe* | NZ80B | AM168029.1 |  |
| *C. aureostipes* var.  *helvetia* | HM592 | KF662214.1 | *R. capillare* | 37A | JF892721.1 |  |
| *C. basinodulosa* | Mad5-1A | MN338955.1 | *R. crispum* | Eden2 | MH762957.1 |  |
| *C. bhumiboliana* | THC11X | HQ141523.1 | *R. cymosum* | Krug15A | MH762958.1 |  |
| *C. bifurcata* | UK5 | AM168084.1 | *R. filiforme* | OH603 | JF892724.1 |  |
| *C. boomerangispora* | K26B | HQ141520.1 | *R. gracile* | TNS-C-183 | AM168078.1 |  |
| *C. canoespora* | Mad14-3C | MN338956.1 | *R. ibericum* | 214rjb | HQ141495.1 |  |
| *C. delicata* | TNS-C-226 | AM168093.1 | *R. maeandriforme* | OH604 | JF892719.1 |  |
| *C. deminutiva* | MexM19A | AM168092.1 | *R. minutum* | 71-2 | AM168051.1 |  |
| *C. exigua* | TNS-C-199 | AM168085.1 | *R. monochasioides* | HAG653 | AM168052.1 |  |
| *C. fasciculata* | SmokOW9A | AM168086.1 | ***R. macrosorus*** | **DM3 3D2** | **PQ677053** |  |
| *C. fasciculata* | SH3 | AM168087.1 | *R. ohioense* | Okla4C | HQ141493.1 |  |
| *C. fasciculoidea* |  | GQ496157.1 | *R. potamoides* | FP1A | AM168069.1 |  |
| *C. fulva* | Krug6-5A | MH762953.1 | *R. reciprocatum* | 38A | JF892718.1 |  |
| *C. granulophora* | CHII-4 | AM168072.1 | *R. reciprocatum* var. *transitum* | OH601 | JF892723.1 |  |
| *C. helicoidea* | Landolt  TH19B | OM677255.1 | *R. stabile* | M12A | MN338957.1 |  |
| *C. macrocarpa* | MGE2 | HQ141519.1 | *R. tenue* | Pan52 | AM168076.1 |  |
| *C. medusoides* | OH592 | AM168088.1 | *R. tenue* | PJ6 | AM168094.1 |  |
| *C. mexicana* | MexTF4B1 | AM168089.1 | *R. tenue* | PR4 | AM168075.1 |  |
| *C. microspora* | TNS-C-38 | AM168090.1 | *Speleostelium  caveatum* | WS695 | AM168077.1 |  |
| *C. minima* | Eden1 | MH762954.1 | *Dictyostelium  ammophilum* | KBK4A | HQ141478.1 |  |
| ***C. densissima*** | **SY1 1F2** | **PQ677055** | *D. aureocephalum* | TNS-C-180 | AM167876.1 |  |
| ***C. densissima*** | **SY3 2F1** | **PQ677056** | *D. aureum* | SL1 | AM168028.1 |  |
| *C. multistipes* | UK26b | AM168070.1 | *D. austroandinum* |  | GQ496158.1 |  |
| *C. myxobasis* | NT2A | HQ141522.1 | *D. barbarae* | 1-5 | MK322959.1 |  |
| *C. parvibrachiata* | Landolt  TH20C | OM677256.1 | *D. barbibulus* | Sweden-4R | JX173878.1 |  |
| *C. parvibrachiata* | Landolt 2019 TH20C | OM677257.1 | *D. brefeldianum* | TNS-C-115 | AM168030.1 |  |
|  |  |  |  |  |  |  |
| *C. parvispora* | OS126 | AM168091.1 | *D. brunneum* | WS700 | AM168031.1 |  |
| *C. protodigitata* | TH18BA | MH745572.1 | *D. capitatum* | 91HO-50 | AM168032.1 |  |
| *C. protumula* | Landolt  TH20A | OM677258.1 | *D. chordatum* |  | GQ496159.1 |  |
| *C. pseudoaureostipes* | TH39A | HQ141518.1 | *D. citrinum* | OH494 | AM168033.1 |  |
| *C. stellata* | SAB7B | AM168081.1 | *D. clavatum* | TNS-C-189 | AM168034.1 |  |
| *C. subdiscoidea* | TH1A | HQ141515.1 | *D. clavatum* | TNS-C-220 | AM168035.1 |  |
| *C. ungulata* | Landolt  TH18B | OM677259.1 | *D. crassicaule* | 93HO-33 | AM168037.1 |  |
| *Acytostelium  amazonicum* | Landolt X | HQ141510.1 | *D. dimigraformum* | AR5b | AM168038.1 |  |
| *A. amazonicum* | HN1B1 | HQ141511.1 | *D. discoideum* | NC4 | AM168071.1 |  |
| *A. anastomosans* | PP1 | AM168115.1 | *D. discoideum* | V34 | AM168039.1 |  |
| *A. digitatum* | OH517 | AM168114.1 | *D. firmibasis* | TNS-C-14 | AM168041.1 |  |
| *A. leptosomum* | 212rjb | HQ141512.1 | *D. gargantuum* |  | GQ496161.1 |  |
| *A. leptosomum* | FG12 | AM168111.1 | *D. giganteum* | WS589 | AM168042.1 |  |
| *A. longisorophorum* | DB10A | AM168109.1 | *D. longigracilis* | 6709C | PQ287291 |  |
| *A. magnisorum* | 08A | HQ141513.1 | *D. longigracilis* | 6709L | PQ287298 |  |
| *A. serpentarium* | SAB3A | AM168113.1 | *D. longigracilis* | 6709M | PQ287299 |  |
| *A. singulare* | FDIB | HQ141514.1 | *D. implicatum* | 93HO-1 | AM168043.1 |  |
| *A. subglobosum* | LB1 | AM168110.1 | ***D. torta*** | **GC2 1E1** | **PQ677054** |  |
| *Rostrostelium  ellipticum* | AE2 | AM168112.1 | *D. insulinativitatis* |  | MK322958.1 |  |
| *Heterostelium  album* | PN500 | AM168104.1 | *D. intermedium* | PJ11 | AM168044.1 |  |
| *H. ampliverticillatum* |  | KP167480.1 | *D. leptosomopsis* | Araucaria 1 | HM159992.1 |  |
| *H. anisocaule* | NZ47B | AM168096.1 | *D. leptosomum* | NZN49A | HQ141480.1 |  |
| *H. arachnoideum* | YA1 | AM168102.1 | *D. longosporum* | TNS-C-109 | AM168048.1 |  |
| *H. asymetricum* | OH567 | AM168097.1 | *D. macrocephalum* | B33 | AM168049.1 |  |
| *H. asymetricum* | HN20C | HQ141503.1 | *D. medium* | TNS-C-205 | AM168050.1 |  |
| *H. australicum* | NB1AP | HQ141508.1 | *D. minimum* | 2794 | MG490369.1 |  |
| *H. boreale* | BSB10A | HQ141499.1 | *D. brefeldianum* | S28b | AM168054.1 |  |
| *H. candidum* | bsb6b | HQ141498.1 | *D. mucoroides*  var. *stoloniferum* | FOII-1 | AM168055.1 |  |
| *H. candidum* |  | AY040337.1 | *D. multiforme* | 4007 | MG490370.1 |  |
| *H. colligatum* | HN13C1 | HQ141505.1 | *D. pseudobrefeldianum* | 91HO-8 | AM168059.1 |  |
| *H. colligatum* | OH538 | AM168098.1 | *D. purpureum* | QSpu1 | FJ424829.1 |  |
| *H. cumulocystum* |  | KP167479.1 | *D. purpureum* | QSpu2 | FJ424839.1 |  |
| *H. equisetoides* | B7JB | AM168099.1 | *D. purpureum* | QSpu23 | FJ424832.1 |  |
| *H. filamentosum* | SU-1 | AM168100.1 | *D. purpureum* | QSpu28 | FJ424836.1 |  |
| *H. flexuosum* | AU4B | HQ141500.1 | *D. purpureum* |  | DQ340386.1 |  |
| *H. gloeosporum* | TCK52 | AM168074.1 | *D. purpureum* | QSpu4 | FJ424826.1 |  |
| *H. granulosum* | MF5A | HQ141502.1 | *D. purpureum* |  | AY040335.1 |  |
| *H. irregularibrachiatum* | Krug6-5B | MH762955.1 | *D. purpureum* | QSpu36 | FJ424828.1 |  |
| *H. lapidosum* |  | KP167477.1 | *D. purpureum* | C143 | AM168060.1 |  |
| *H. luridum* | LR-2 | AM168101.1 | *D. purpureum* | WS321 | AM168061.1 |  |
| *H. migratissimum* |  | KP167481.1 | *D. purpureum* | cavender | HQ141481.1 |  |
| *H. multicystogenum* | AS2 | HQ141506.1 | *D. purpureum* var. *pseudosessile* | MR273 (4637) | MH280022.1 |  |
| *H. oculare* |  | HQ141497.1 | *D. purpureum* var. *pseudosessile* | MR273 (4446) | MH280023.1 |  |
| *H. oculare* | DB4B | AM168079.1 | *D. quercibrachium* | NZ201B | HQ141479.1 |  |
|  |  |  | *D. robusticaule* | 5729-bai-2021 | MW931857.1 |  |
| *H. pallidum* | PPHU8 | EU004605.1 | *D. robusticaule* | 5729-huang-2021 | MW931856.1 |  |
| *H. pallidum* | TNS-C-98 | AM168103.1 |  |  |  |  |
| *H. parvimigratum* |  | KP167483.1 | *D. macrosoriobrevipes* | 6718S1-2B | PQ304778 |  |
| *H. plurimicrocystogenum* |  | KP167475.1 | *D. robustum* | TNS-C-219 | AM168064.1 |  |
| *H. pseudocandidum* | TNS-C-91 | AM168107.1 | *D. rosarium* | M45 | AM168065.1 |  |
| *H. pseudocolligatum* |  | KP167474.1 | *D. septentrionale* | IY49 | AM168066.1 |  |
| *H. pseudoplasmodiofascium* |  | KP167482.1 | *D. septentrionale* | AK2 | AM168067.1 |  |
| *H. pseudoplasmodiomagnum* |  | KP167472.1 | *D. sphaerocephalum* | GR11 | AM168068.1 |  |
| *H. racemiferum* |  | KP167476.1 | *D. valdivianum* |  | GQ496155.1 |  |
| *H. radiatum* | M26B | MN338953.1 | *D. brevicaule* | SMA |  |  |
| *H. rotatum* | QC2C | HQ141501.1 | *Polysphondylium  fuscans* | Sweden-11D | JX173877.1 |  |
| *H. stolonicoideum* | K12A | HQ141507.1 | *P. laterosorum* | AE4 | AM168046.1 |  |
| *H. tenuissimum* | TNS-C-97 | AM168105.1 | *P. patagonicum* |  | GQ496156.1 |  |
| *H. tenuissimum* |  | AY040339.1 | *P. violaceum* | 209 | HQ141486.1 |  |
| *H. tikalense* | OH595 | AM168106.1 | *P. violaceum* | P6 | AM168108.1 |  |
| *H. tikalense* | HN1C1 | HQ141509.1 | *P. acuminatum* | OH500 SML |  |  |
| *H. unguliferum* |  | KP167473.1 | ***P. sparsiramus*** | **SY1 1F4** | PQ677059 |  |
| *H. versatile* | Mad52 | MN338954.1 | ***P. sparsiramus*** | **SY3 1B1** | PQ677058 |  |
|  |  |  | ***P. sparsiramus*** | **SY3 1C1** | PQ677057 |  |
| *H. violaceotypum* |  | KP167478.1 | ***P. sparsiramus*** | **SY3 1D1** | PQ677060 |  |
|  |  |  | ***P. sparsiramus*** | **SY3 1D2** | PQ677062 |  |
| *H. recretum* | 5756-1-17 | MW857293.1 | ***P. sparsiramus*** | **SY3 1F1** | PQ677061 |  |
|  |  |  | ***P. sparsiramus*** | **GY1 2B2** | PQ686236 |  |
| *H. multibrachiatum* | 5916lun | MN752217.1 | *Coremiostelium  polycephalum* | Landolt #1130 SS3B | HQ141488.1 |  |
| *H. naviculare* | JC SMA |  |  |  |  |  |
| *Tieghemostelium angelicum* | 38B0 | JF892716.1 | *Co. polycephalum* | Landolt #2132 B-9c | HQ141489.1 |  |
| *T. dumosum* | OH602 | JF892722.1 |  |  |  |  |
| *T. lacteum* |  | AM168045.1 | *Co. polycephalum* | Landolt #1675 GUAM | HQ141490.1 |  |
| *T. menorah* | M1 | AM168073.1 |  |  |  |  |
| *T. montium* | 57a | JF892717.1 | *Co. polycephalum* | MY1-1 | AM168056.1 |  |
| *T. simplex* | OH598 | JF892720.1 | ***Co. viridiflava*** | **DM1 1F1** | **PQ677052** |  |
| *T. unicornutum* | OH599 | JF892725.1 | *Synstelium polycarpum* | VE1b | AM168057.1 |  |
| *Hagiwaraea coeruleostipes* | CRLC53B | AM168036.1 | *S. polycarpum* | OhioWILDS | AM168058.1 |  |
|  |  |  | *Physarum polycephalum* |  | X13160.1 |  |

Newly generated sequences are shown in bold.
